# Predictive Performance of Hematological Parameters for Acute Respiratory Distress Syndrome in Hospitalized Pregnant and Postpartum Women With COVID-19

**DOI:** 10.7759/cureus.111714

**Published:** 2026-06-29

**Authors:** Renata I Araujo, Eloisa H Kubiszeski, Marcial F Galera, Anselmo V Carmo, Maria Aparecida M Carmo, Cor J Fontes

**Affiliations:** 1 Faculty of Medicine, Post-Graduate Program in Health Science, Universidade Federal de Mato Grosso, Cuiabá, BRA; 2 Obstetrics and Gynecology, Hospital Universitário Júlio Müller, Universidade Federal de Mato Grosso, Cuiabá, BRA; 3 Pediatrics, Hospital Universitário Júlio Müller, Universidade Federal de Mato Grosso, Cuiabá, BRA; 4 Infectious Diseases, Hospital Universitário Júlio Müller, Universidade Federal de Mato Grosso, Cuiabá, BRA

**Keywords:** acute respiratory distress syndrome, covid-19, hematological parameters, postpartum period, pregnancy, white blood cell parameters

## Abstract

Introduction: Early identification of risk factors for acute respiratory distress syndrome (ARDS) in patients with coronavirus disease 2019 (COVID-19) is essential to guide preventive interventions. This study aimed to evaluate the predictive performance of hematological parameters measured at hospital admission for ARDS in pregnant and postpartum women with COVID-19. Materials and methods: This retrospective cohort study included all pregnant and postpartum women hospitalized with COVID-19 between March 2020 and October 2021. Demographic, clinical, and laboratory data were extracted from medical records. Total white blood cell (WBC), neutrophil, lymphocyte, and monocyte counts, as well as the neutrophil-to-lymphocyte ratio (NLR) and monocyte-to-lymphocyte ratio (MLR), were evaluated using receiver operating characteristic curve analysis, and the area under the curve (AUC) was calculated to assess their predictive performance for ARDS in these groups. Results: A total of 126 patients were included, of whom 75.4% were aged 26-40 years. Overweight (37.8%) and obesity (45.9%) were the most frequent comorbidities. ARDS developed in 43.6% of patients, and 68.3% required invasive mechanical ventilation. Patients with ARDS had significantly higher WBC and neutrophil counts and lower lymphocyte counts (all p < 0.001). Both NLR and MLR were significantly associated with ARDS (p < 0.001). NLR showed the highest predictive performance for ARDS (AUC = 0.86) and a sensitivity of 81.5%. MLR demonstrated moderate predictive performance (AUC = 0.73) with a sensitivity of 63.0%. Conclusions: Hematological parameters, particularly the NLR and MLR, measured at hospital admission may be useful for assessing the risk of ARDS in pregnant and postpartum women with COVID-19. Further studies are needed to establish the predictive performance of these parameters for ARDS in this population.

## Introduction

Severe manifestations of coronavirus disease 2019 (COVID-19) frequently progress to acute respiratory distress syndrome (ARDS), a complication strongly associated with increased mortality. Previous studies have estimated that nearly one-third of hospitalized patients with COVID-19 develop ARDS, and respiratory failure accounts for a substantial proportion of ARDS-related deaths [1]. By 2024, more than 776 million confirmed COVID-19 cases and approximately 7 million deaths had been reported worldwide, including nearly 38.9 million confirmed cases and more than 713,000 deaths in Brazil [2,3].

Pregnancy has been recognized as a risk factor for unfavorable clinical outcomes in women with COVID-19. During the early stages of the pandemic, mortality among pregnant women was reported to be markedly higher than that observed in the general population [4,5]. Reduced access to healthcare services and delays in diagnosis, partially related to social distancing measures, may have contributed to these adverse outcomes [6]. Physiological and immunological adaptations occurring during pregnancy may also influence disease severity. The first and third trimesters are characterized by enhanced inflammatory activity, which may favor viral pathogenicity and contribute to severe clinical manifestations [7,8]. Leukocytosis is a common physiological finding during pregnancy and is primarily driven by an increase in neutrophil counts [9]. These hematologic changes are considered adaptive responses associated with hormonal regulation and maternal immune tolerance mechanisms required for fetal development [10].

Infection with severe acute respiratory syndrome coronavirus 2 (SARS-CoV-2) triggers systemic inflammatory responses that may lead to immune dysregulation and cytokine-mediated tissue injury, thereby contributing to the development of ARDS. Lymphopenia is frequently observed in severe COVID-19 and has been associated with poor clinical outcomes [11]. Likewise, neutrophilia reflects an exaggerated inflammatory response and has consistently been associated with increased disease severity and mortality [12]. The pro-inflammatory immune environment observed during pregnancy may further increase susceptibility to severe respiratory complications, particularly during the first and third trimesters [13].

Hematological parameters, particularly the neutrophil-to-lymphocyte ratio (NLR) and monocyte-to-lymphocyte ratio (MLR), have increasingly been investigated as markers of systemic inflammatory activity [14]. Elevated NLR values have been associated with severe outcomes in several inflammatory and infectious conditions, reflecting the intensity of the host inflammatory response [14-16]. Because these parameters are readily obtained from routine blood tests, they may serve as practical, accessible tools for early clinical assessment.

Although previous studies conducted mainly in China and Europe have suggested prognostic utility of NLR and MLR in COVID-19 [12,15], evidence from Brazil remains scarce, especially among pregnant and postpartum women. Early recognition of laboratory markers associated with severe disease may support timely clinical decision-making and improve patient management. Therefore, the present study aimed to investigate the predictive performance of hematological parameters measured at hospital admission for identifying ARDS among pregnant and postpartum women hospitalized with COVID-19.

## Materials and methods

Study design and setting

A retrospective cohort study was conducted among pregnant and postpartum women hospitalized with COVID-19 at Júlio Müller University Hospital, Cuiabá, Brazil, between March 2020 and October 2021. During the study period, the hospital served as a regional referral center for pregnant and postpartum women with suspected or confirmed SARS-CoV-2 infection who met clinical criteria for hospital admission. Therefore, the study population was restricted to hospitalized patients and does not represent all pregnant and postpartum women with SARS-CoV-2 infection in the community.

Study participants

All pregnant and postpartum women hospitalized with COVID-19 during the study period were included in the analysis. Although the study was initially designed to include only pregnant women, 10 participants experienced pregnancy termination due to COVID-19-related complications shortly before study enrollment and were therefore classified as postpartum women. Only pregnant and postpartum women with laboratory-confirmed COVID-19 were included in the study. SARS-CoV-2 infection was confirmed by at least one positive diagnostic test, including reverse transcription polymerase chain reaction (RT-PCR), antigen testing, or serological testing, according to the diagnostic methods available during the study period.

Data collection

Demographic, clinical, and laboratory data were obtained from medical records. Variables included age, confirmed COVID-19 diagnosis, comorbidities, need for intensive care, and clinical complications. Hematological parameters obtained at hospital admission included total white blood cell (WBC), neutrophil, monocyte, and lymphocyte counts, which were used to calculate the NLR and MLR.

Study outcome

The primary outcome was the incidence of ARDS, defined according to the Berlin criteria. ARDS was characterized as an acute diffuse lung injury occurring in patients with a predisposing risk factor and meeting the following criteria: (1) onset within 1 week of a known clinical insult or new/worsening respiratory symptoms; (2) bilateral opacities on chest imaging not fully explained by pleural effusions, lobar or lung collapse, or pulmonary nodules; (3) respiratory failure not fully explained by cardiac failure or fluid overload; and (4) hypoxemia defined by a PaO₂/FiO₂ ratio below specified thresholds with a minimum positive end-expiratory pressure ≥5 cm H₂O [10].

Statistical analysis

Descriptive statistics were expressed as means ± standard deviation (SD) for continuous variables and proportions for categorical variables. Variations in sample size across analyses were due to missing data for some variables. Given the low proportion of missing data, incomplete cases were excluded from the analyses. The Mann-Whitney U test was used to compare hematological parameters between patients with and without ARDS. Receiver operating characteristic (ROC) curves were constructed to evaluate the predictive performance of the hematological parameters, with higher area under the curve (AUC) values indicating greater discriminatory ability for ARDS. Sensitivity and specificity at the optimal cutoff were calculated, along with their corresponding 95% confidence intervals, using exact binomial methods. The optimal threshold for each hematological parameter was identified by maximizing Youden's J statistic. The corresponding 95% confidence interval was estimated using nonparametric bootstrap resampling with 1,000 replications. A p-value < 0.05 was considered statistically significant. All statistical analyses were performed using Stata version 12 (StataCorp, College Station, TX, USA).

Ethical considerations

This study was conducted in accordance with the principles of the Declaration of Helsinki (2008). The study protocol was approved by the Research Ethics Committee of Júlio Müller University Hospital (approval no. 4.622.295). Written informed consent was obtained at hospital admission from all eligible pregnant and postpartum women. The informed consent form authorized the use of clinical and laboratory information contained in their medical records for research purposes. For participants younger than 18 years of age, written informed consent was obtained from their parents or legal guardians.

## Results

A total of 126 women were included in the analysis, comprising 116 pregnant women and 10 postpartum women. Because the study was conducted at a referral hospital for COVID-19-related obstetric admissions, the cohort included a substantial proportion of patients with severe disease. Participants’ residences were similarly distributed between Cuiabá's metropolitan region (50.8%) and the state's interior regions (49.2%). The mean age was 29.7 ± 6.1 years. Most participants were aged 26-40 years (75.4%), followed by those aged 19-25 years (20.6%) and adolescents aged 12-18 years (4.0%). Reverse transcription quantitative polymerase chain reaction (RT-qPCR) was the most frequently used diagnostic test for COVID-19 (42.0%), followed by serological tests for IgG and IgM antibodies (31.0%) and nasopharyngeal antigen tests (27.0%). Comorbidities were present in 59.5% of patients, with overweight (37.8%) and obesity (45.9%) being the most prevalent, followed by diabetes mellitus (19.0%) and systemic arterial hypertension (17.5%). Asthma and autoimmune diseases were reported in 4.8% and 4.0% of cases, respectively (Table 1).

**Table 1:** Demographic and clinical characteristics of pregnant/postpartum women admitted for COVID-19 in a university hospital in the Central Brazil from March 2020 to October 2021 * The occurrence of more than one comorbidity or complication in the same patient. Variation in n occurred due to a lack of information for the respective variable. SD: standard deviation, COVID-19: coronavirus disease 2019, RT-qPCR: reverse transcription quantitative polymerase chain reaction, IgM: immunoglobulin M, IgG: immunoglobulin G, ARDS: acute respiratory distress syndrome

Characteristics		n	%
Region of residence	State capital	64	50.8
	Contryside	62	49.2
Age (years)	12-18	5	4
	19-25	26	20.6
	26-40	95	75.4
	Mean (SD): 29.7 (6.1)	-	-
COVID-19 confirmation	RT-qPCR	53	42
	Antigen test	34	27
	IgM/IgG antibody test	39	31
Comorbidity*	Obesity	45	45.9
	Overweight	37	37.8
	Diabetes	24	19
	Arterial hypertension	22	17.5
	Asthma	6	4.8
	Autoimmune disease	5	4
	Cardiopathy	3	2.4
	Hypothyroidism	3	2.4
	Other	12	9.4
	Total with comorbidity	75	59.5
Need for intensive care	Yes	86	68.3
	No	40	31.7
ARDS occurrence	Yes	55	43.6
	No	71	56.4
Other complications*	Non-severe respiratory failure	11	8.7
	Stroke or cerebrovascular accident	1	0.8
	Acute pulmonary edema	15	11.9
	Pulmonary thromboembolism	12	9.5
	Sepsis	15	11.9
	Surgical site infection	9	7.1
	Total with other complications	58	46
Outcome	Discharge	118	93.7
	Death	8	6.3

Overall, 68.3% (n = 86) of the women required admission to the intensive care unit (ICU), and 43.6% (n = 55) developed ARDS. Other complications, including non-severe respiratory failure, stroke, acute pulmonary edema, thromboembolic events, and sepsis, were reported in 46.0% of patients. After a mean hospital stay of 20 ± 15.9 days, 93.6% (n = 118) were discharged, whereas eight women (6.3%) died (Table 1). Of these deaths, five occurred during pregnancy and three during the postpartum period.

Mean leukocyte and neutrophil counts were significantly higher in patients with ARDS than in those without ARDS, with mean values of 14,531 ± 5,826 cells/mm³ and 12,799 ± 5,263 cells/mm³ (U = 976.5; p < 0.001 and U = 855.5; p < 0.001), respectively. In contrast, mean lymphocyte counts were significantly lower in patients with ARDS (1,036 ± 486 cells/mm³; U = 1,145.5; p < 0.001). Although monocyte counts were higher in the ARDS group, the difference did not reach statistical significance (U = 1,653.5; p = 0.141). Both NLR and MLR were significantly elevated in patients with ARDS. The mean NLR was 21.5 ± 55.6 in the ARDS group, compared with 6.3 ± 4.2 in the non-ARDS group (U = 531; p < 0.001). Similarly, the mean MLR was 1.1 ± 4.4 in patients with ARDS versus 0.3 ± 0.2 in those without ARDS (U = 1,027; p < 0.001) (Table 2).

**Table 2:** Analysis of the association between hematological parameters and the incidence of ARDS among pregnant/postpartum women admitted for COVID-19 in a university hospital in the Central Region of Brazil from March 2020 to October 2021 * It was not possible to obtain all the data for one of the patients, ** non-parametric U Mann-Whitney test SD: standard deviation, ARDS: acute respiratory distress syndrome, COVID-19: coronavirus disease 2019

Hematological parameters	Total of patients*	ARDS		p**
		Yes	No	-
		Mean (SD)	Mean (SD)	-
Leukocytes/mm^3^	125	14,531 (5,826)	9,856 (4,202)	<0.001
Neutrophil/mm^3^	126	12,799 (5,263)	7,957 (3,814)	<0.001
Monocyte/mm^3^	126	526 (320)	442 (262)	0.141
Lymphocyte/mm^3^	125	1,036 (486)	1,545 (823)	<0.001
Neutrophil/lymphocyte ratio	125	21.5 (55.6)	6.3 (4.2)	<0.001
Monocyte/lymphocyte ratio	125	1.1 (4.4)	0.3 (0.2)	<0.001

**Figure 1:**
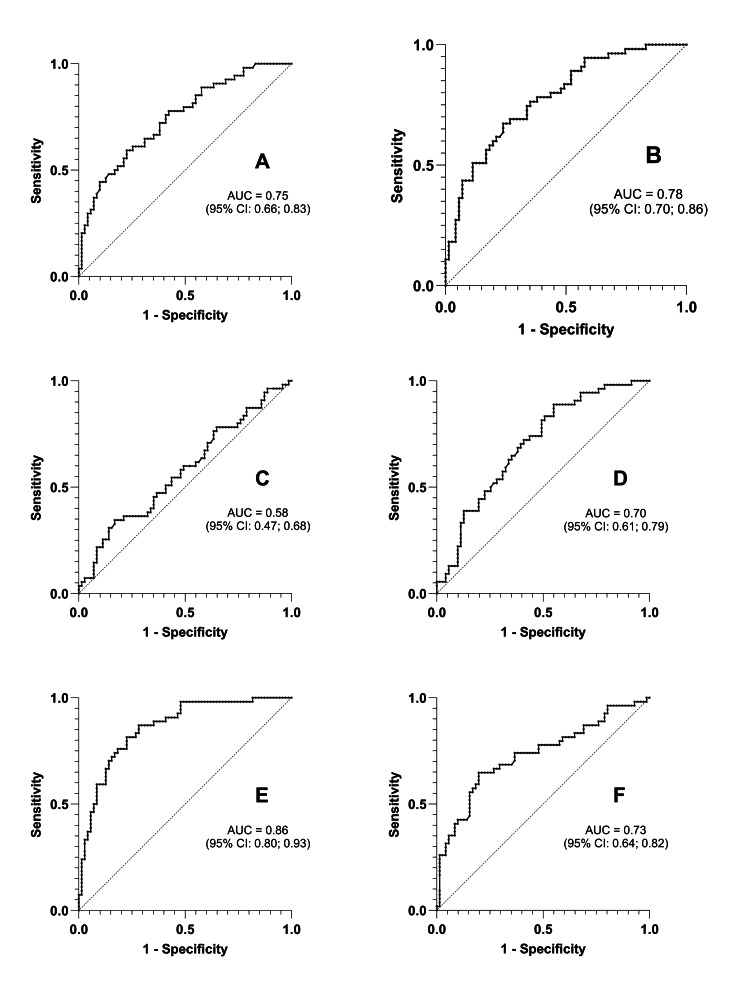
ROC curves demonstrating the predictive performance of hematological parameters for ARDS among pregnant and postpartum women hospitalized with COVID-19 in a university hospital in Central Brazil between March 2020 and October 2021 (A) WBC count - cutoff: 12,650 cells/mm³ (95% CI: 7,880-15,710); sensitivity: 59.3% (95% CI: 45.0-72.4%); specificity: 77.4% (95% CI: 66.0-86.5%). (B) Neutrophil count – cutoff: 10,144 cells/mm³ (95% CI: 6,229-13,313); sensitivity: 66.7% (95% CI: 52.5-78.9%); specificity: 76.0% (95% CI: 64.4-85.4%). (C) Monocyte count - cutoff: 628 cells/mm^3^ (95% CI: 233-759); sensitivity: 35.2% (95% CI: 22.7-49.4%); specificity: 83.1% (95% CI: 72.3-91.0%). (D) Lymphocyte count - cutoff: 1478 cells/mm^3^ (95% CI: 773-1894); sensitivity: 88.9% (95% CI: 77.4-95.8%); specificity: 43.7% (95% CI: 31.9-55.9%). (E) NLR - cutoff: 7.82 (95% CI: 5.78-9.78); sensitivity: 81.5% (95% CI: 68.6-90.7%); specificity: 76.0% (95% CI: 64.4-85.4%). (F) MLR - cutoff: 0.36 (95% CI: 0.33-0.46); sensitivity: 64.0% (95% CI: 48.7-75.7%); specificity: 80.3% (69.1-88.8%). ROC: receiver operating characteristic, ARDS: acute respiratory distress syndrome, COVID-19: coronavirus disease 2019, WBC: white blood cell, CI: confidence interval, NLR: neutrophil-to-lymphocyte ratio, MLR: monocyte-to-lymphocyte ratio

ROC curve analysis demonstrated good predictive performance of several hematological parameters for ARDS. The highest AUC values were observed for NLR (0.86), neutrophil count (0.78), WBC count (0.75), MLR (0.73), and lymphocyte count (0.70), indicating that NLR has superior predictive performance. The optimal cut-off points for each parameter yielded sensitivities ranging from 35.2% (monocyte count) to 81.5% (NLR) and specificities ranging from 76.0% (neutrophil count) to 89.0% (lymphocyte count) (Table 3, Figure 1A-1F).

**Table 3:** Cutoff points of hematological parameters with potential diagnostic value for severe ARDS among pregnant/postpartum women admitted for COVID-19 in a university hospital in the Central Brazil from March 2020 to October 2021 ARDS: acute respiratory distress syndrome, COVID-19: coronavirus disease 2019

Hematological parameters	Cutoff point	J-index (Youden)	Sensitivity	Specificity	Accuracy
			%	%	%
WBC/mm^3^	12620	0.367	59	77	68
Neutrophil/mm^3^	10048	0.433	67	76	72
Monocyte/mm^3^	626	0.176	35	83	59
Lymphocyte/mm^3^	1470	0.340	45	89	67
Neutrophil/lymphocyte ratio	7.864	0.589	81	77	79
Monocyte/lymphocyte ratio	0.364	0.451	65	80	73

## Discussion

In the present study, elevated WBC and neutrophil counts, lymphopenia, and increased NLR and MLR demonstrated satisfactory predictive performance for ARDS in pregnant and postpartum women hospitalized with COVID-19. Their low cost, rapid availability, and widespread accessibility make these laboratory parameters particularly useful for routine clinical evaluation. To our knowledge, this investigation is among the first to evaluate the predictive performance of admission hematological parameters for identifying pregnant and postpartum women at increased risk of developing COVID-19-associated ARDS.

This study has several strengths. First, it focused on a population that remains underrepresented in the COVID-19 literature, namely pregnant and postpartum women hospitalized with SARS-CoV-2 infection. Second, the study was conducted in Brazil, where data regarding the predictive performance of hematological biomarkers in obstetric patients with COVID-19 remain limited. Third, hematological parameters were assessed at hospital admission, reflecting information readily available to clinicians during the initial evaluation. Additionally, the cohort included a substantial proportion of patients who developed ARDS, allowing a comprehensive assessment of biomarker performance in a population at high risk for severe respiratory complications. Finally, because pregnancy is characterized by unique physiological and immunological adaptations that influence leukocyte dynamics, our findings provide further evidence for the clinical utility of leukocyte-derived biomarkers in this population. To our knowledge, this is one of the first Brazilian studies to evaluate the discriminatory performance of admission hematological parameters for ARDS among hospitalized pregnant and postpartum women with COVID-19.

Pregnancy is associated with several alterations in neutrophil biology, including delayed apoptosis, reduced chemotaxis, and impaired respiratory burst activity, all of which may contribute to enhanced inflammatory responses [12]. Although neutrophils in normal pregnancy are not typically activated, their ability to generate reactive oxygen species appears to be altered [12]. These changes are believed to represent compensatory adaptations of innate immunity during the attenuation of antigen-specific immune responses required for fetal tolerance [13].

Elevated WBC counts during pregnancy may represent either physiological adaptation or evidence of severe infection. Under normal conditions, leukocyte counts gradually rise throughout pregnancy due to physiological inflammatory changes and immune modulation. Previous studies have shown that leukocyte counts may reach upper reference limits of 13.2 × 10³/µL at the 95th percentile and 15.9 × 10³/µL at the 99th percentile in healthy pregnant women [14]. However, in infectious conditions, leukocyte counts exceeding pregnancy-specific reference intervals, especially values above 15 × 10³/µL, may indicate severe infection and have been associated with adverse clinical outcomes [6].

The demographic and clinical characteristics observed in this cohort are consistent with previous reports involving pregnant women with COVID-19 [15]. Pregnancy itself is considered a risk factor for severe COVID-19-related complications [16], and comorbidities such as obesity, diabetes mellitus, hypertension, and asthma were common among the participants, similar to findings reported in Brazilian and international studies [17]. Although mortality was relatively low in the present investigation, deaths occurred predominantly among patients requiring ICU admission and mechanical ventilation due to ARDS, which is consistent with prior studies describing increased mortality among pregnant women with severe COVID-19 complications [17].

Higher WBC and neutrophil counts and lower lymphocyte counts at hospital admission were significantly associated with the development of ARDS in the present study, whereas monocyte counts were not. These hematologic abnormalities are frequently reported in severe COVID-19, and leukocytosis has often been associated with secondary bacterial infection and unfavorable clinical evolution [18].

Neutrophils are central mediators of innate immune responses and may contribute to tissue injury and multiple organ dysfunction in severe COVID-19 [19]. Previous investigations have demonstrated a predominance of neutrophilia among patients presenting severe complications, including ARDS [20]. Lymphopenia has also consistently been identified as a marker of poor prognosis in COVID-19. Several mechanisms have been proposed to explain this finding, including direct viral effects on lymphocytes and cytokine-mediated immune dysfunction [21].

NLR has been widely used as an indicator of the interaction between inflammatory activation and immune dysfunction in infectious diseases. Higher NLR values generally reflect more intense systemic inflammatory responses [22,23]. Dynamic changes in NLR during severe infections have been associated with clinical deterioration and unfavorable outcomes, reinforcing the utility of this index as a prognostic predictor [24,25]. Elevated NLR values have additionally been reported in severe respiratory syncytial virus infection, sepsis, and leprosy reactions [23,26,27].

Although monocyte counts alone were not significantly associated with ARDS in this cohort, MLR demonstrated satisfactory predictive performance, reaching 65% sensitivity at a cutoff value of 0.36. Despite the limited use of MLR in COVID-19 prognostic assessment, previous studies have reported an association between MLR and ICU admission and early mortality [28]. Although external validation is still lacking, our findings are consistent with observations reported in other patient populations hospitalized with COVID-19 [24,29,30].

Several limitations should be considered when interpreting these findings. The study was conducted at a single referral hospital over a limited time period, which may restrict external generalizability. The small number of postpartum women prevented subgroup analyses comparing pregnant and postpartum patients separately. Additionally, the retrospective design may have introduced information bias related to incomplete or inaccurately recorded medical data. Another important limitation is that the cutoff values identified in this study were empirically derived from ROC curve analysis using Youden’s J index and, therefore, should be interpreted as exploratory thresholds that require external validation before routine clinical implementation. An additional limitation is that the study was conducted at a regional referral center for pregnant and postpartum women with COVID-19 who met clinical criteria for hospital admission. Consequently, the cohort included a high proportion of patients with severe disease, which may limit the generalizability of the findings to outpatient populations and to hospitalized patients with less severe clinical presentations. Finally, the present analysis was exploratory and focused on the univariable discriminatory performance of individual hematological parameters. The reported AUC values were not adjusted for potential confounding factors, nor were they compared with multivariable clinical prediction models. Therefore, the findings should be interpreted as evidence of discriminatory ability rather than as a validated prognostic model. Future studies should evaluate the incremental predictive value of these hematological parameters within adjusted multivariable models. Additionally, multicenter investigations involving larger populations and longitudinal follow-up are necessary to confirm the reproducibility and generalizability of these findings.

Few laboratory tools are currently available to assist in the early identification of pregnant women at risk of severe COVID-19 complications. The present findings suggest that inexpensive and readily available hematological parameters may provide useful predictive information regarding ARDS risk in pregnant and postpartum women hospitalized with COVID-19. These parameters may contribute to rapid risk stratification and support clinical decisions regarding monitoring intensity, triage, and level of care.

## Conclusions

Admission hematological parameters, particularly the NLR and MLR, demonstrated satisfactory predictive performance in identifying pregnant and postpartum women hospitalized with COVID-19 at increased risk of developing ARDS. Elevated WBC and neutrophil counts, lymphopenia, and increased NLR and MLR were associated with ARDS occurrence, with NLR showing the best discriminatory performance among the evaluated parameters. Given their low cost, widespread availability, and rapid availability of results, these hematological parameters may serve as useful tools for early risk stratification and clinical decision-making in hospitalized obstetric patients, particularly in resource-limited settings. However, because this study was conducted in a referral center with a high proportion of severe cases, prospective multicenter studies involving broader patient populations are needed to externally validate these findings and establish their applicability across different clinical settings.
